# Role of purinergic signalling in obesity-associated end-organ damage: focus on the effects of natural plant extracts

**DOI:** 10.3389/fendo.2023.1181948

**Published:** 2023-07-05

**Authors:** Hangxiu Che, Yaqun Wang, Jinhui Lao, Yixin Deng, Chirui Xu, Hanxiao Yin, Zheng Tang, Yonghong Huang, Hong Xu

**Affiliations:** ^1^ Department of Physiology, Jiangxi Medical College of Nanchang University, Nanchang, Jiangxi, China; ^2^ Joint Program of Nanchang University and Queen Mary University of London, Jiangxi Medical College of Nanchang University, Nanchang, Jiangxi, China; ^3^ Basic Medicine, Jiangxi Medical College of Nanchang University, Nanchang, Jiangxi, China; ^4^ Huankui Academy, Jiangxi Medical College of Nanchang University, Nanchang, Jiangxi, China; ^5^ The Second Clinical Medicine, Jiangxi Medical College of Nanchang University, Nanchang, Jiangxi, China; ^6^ Department of Pathophysiology, Jiangxi Medical College of Nanchang University, Nanchang, Jiangxi, China

**Keywords:** obesity, purinergic signalling, ATP, P1 receptor, P2 receptor

## Abstract

Obesity has become one of the major public health problems in both the developing and developed countries. Recent studies have suggested that the purinergic signalling is involved in obesity-associated end-organ damage through purine P1 and P2 receptors. In the search for new components for the treatments of obesity, we and other researchers have found much evidence that natural plant extracts may be promising novel therapeutic approaches by modulating purinergic signalling. In this review, we summarize a critical role of purinergic signalling in modulating obesity-associated end-organ damage, such as overhigh appetite, myocardial ischemia, inflammation, atherosclerosis, non-alcoholic fatty liver disease (NAFLD), hepatic steatosis and renal inflammation. Moreover, we focus on the potential roles of several natural plant extracts, including quercetin, resveratrol/trans-resveratrol, caffeine, evodiamine and puerarin, in alleviating obesity-associated end-organ damage via purinergic signalling. We hope that the current knowledge of the potential roles of natural plant extracts in regulating purinergic signalling would provide new ideas for the treatment of obesity and obesity-associated end-organ damage.

## 1 Introduction

Obesity, characterized by excessive or abnormal fat accumulation, represents a common metabolic disorder in the world (1). The onset and development of obesity have significant impacts on the end-organ damage, leading to severe complications such as neuro-inflammation (2), overhigh appetite (3), myocardial ischemia (4), atherosclerosis (3), non-alcoholic fatty liver disease (NAFLD) (3) and renal damage (5). However, few therapeutic strategies are available for the long-term treatment of obesity, with some of them largely ineffective (6). It is urgently needed to find new directions for the effective treatment strategies.

The purinergic system includes adenine nucleosides (adenosine), nucleotides including adenosine 5′-triphosphate (ATP) and adenosine diphosphate (ADP), purine receptors (P1 and P2 receptors) and ecto-enzymes (3) (**Table 1**). P1 and P2 receptors are two kinds of receptors responding to extracellular purines. ATP can be released to the extracellular environment, acting as a physiological extracellular messenger or as a danger signal in pathophysiological situations (7). For the P1 receptors, it can be categorized into four subtypes, including adenosine A1 receptor (A1R), adenosine A2A receptor (A2AR), adenosine A2B receptor (A2BR) and adenosine A3 receptor (A3R) (7). They are all G-protein coupled receptors. Among them, A2A and A2B receptors couple to stimulatory Gi, Gq and Go protein, which increases the intracellular cyclic adenosine phosphate (cAMP) via activating adenylyl cyclase (AC) (8). But adenosine A1 and A3 receptors preferentially couple to inhibitory Gi or Gq/11 protein and downregulate the activities of AC (7). For the P2 receptors, it can be categorized into two main groups based on molecular structure and second messenger systems, namely purinergic P2X and P2Y receptors (7). The P2X receptors have seven subtypes (P2X1R-P2X7R). The P2X receptors are all ATP ligand-gated ion channel receptors and activated by ATP (9). By binding to ATP, P2X receptors trigger conformational changes and open ion channels, facilitating the influx of Na^+^ and Ca^2+^ ions as well as the efflux of K^+^ ion (9). The P2Y receptors (P2Y1R, P2Y2R, P2Y4R, P2Y6R, P2Y11R, P2Y12R, P2Y13R and P2Y14R) are identified and characterized as metabotropic G protein-coupled receptors (8). They are activated by a group of nucleotides and nucleotide sugars (ATP, ADP, UTP, UDP, UDP-glucose) (8). P2YR1, P2YR2, P2YR4, and P2YR6 activate Gq/G11 and phospholipase C-β to increase intracellular Ca^2+^ level. P2YR12, P2YR13, and P2YR14 corporate with Gi, leading to the inhibition of AC and the reduction of cAMP level (8). P2YR11 increases intracellular Ca^2+^ and cAMP via corporation with both Gq and Gs (8). Moreover, ecto-enzymes are responsible for the metabolism of nucleotides and nucleosides. For example, ectonucleoside triphosphate diphosphohydrolase (ENTPDase) converts ATP or ADP to AMP (8); ecto-5’-nucleotidase is responsible for dephosphorylating AMP to adenosine (10); ectoadenosine deaminase (EADA) converts adenosine to inosine. These ecto-enzymes play vital roles in the regulation of agonist concentration of P1 and P2 receptors and thus may be involved in the pathological processes including obesity.

**Table 1:** The components of purinergic system.

Components	Subtype	Agonist
P1 receptors	A1R	Adenosine
	A3R	
	A2AR	
	A2BR	
P2X receptors	P2X1R	ATP
	P2X2R	
	P2X3R	
	P2X4R	
	P2X5R	
	P2X6R	
	P2X7R	
P2Y receptors	P2Y1R	ATP, ADP, UTP, UDP, UDP-glucose
	P2Y2R	
	P2Y4R	
	P2Y6R	
	P2Y11R	
	P2Y12R	
	P2Y13R	
	P2Y14R	
Ecto-nucleotidases	NTPDase	
	Ecto-5’-nucleotidase	
	Ectoadenosine deaminase	

Several lines of evidence have suggested the link between purinergic signalling and obesity-associated end-organ damage. Purinergic receptors, including P1 receptors, P2X and P2Y receptors, are widely expressed in obesity-associated end-organs or systems, such as adipose tissue, nerve system, cardiovascular system, liver and renal, and the change in their expressions and/or activities occurs in pathological obesity model (3). Besides the purinergic receptors, other components of purinergic signalling, including adenosine, ATP and ectonucleotides, are also suggested to be pathologically deregulated in obesity (9, 11). Such changes of purinergic signalling may modulate the downstream molecules and hold a potential pathophysiological role in the damage of multiple organs in obesity.

Over the last decade, great efforts have been made to search for natural compounds alleviating end-organ damage induced by obesity. Our studies and the studies of other groups (7, 12–17) have suggested that natural plant extracts have the potential to regulate purinergic tuning of pathophysiological obesity processes, which may provide new treatments for the end-organ damage in obesity. In view of this, on one hand, we emphasize the current knowledge of potential roles of purinergic signalling in obesity-associated end-organ damage, and on the other hand, we summarize the potential roles of natural plant extracts in the treatment of obesity and its associated end-organ damage. This review improves our understanding of the effect of purinergic signalling on obesity-associated end-organ damage. Moreover, our review contributes to the understanding of the supplement of natural plant extracts as new non-toxic approaches for the treatment of obesity-associated end-organ damage via purinergic signalling.

## 2 Purinergic signalling and obesity-associated end-organ damage

### 2.1 Purinergic signalling and adipose tissue in obesity

Adipose tissue is a significant endocrine organ, being an essential regulator of adipogenesis, lipolysis and energy homeostasis (18). The expression of most P1 and P2 receptor subtypes was indicated in adipose tissue (1). Furthermore, functional analyses via pharmacological intervention and/or transgenic animals for purinergic receptors have shown an essential role of purinergic signalling in adipogenesis, adipogenic differentiation and inflammatory response (1).

P1 receptors are known to be pivotal in modulating energy storage (1). A1R is abundantly expressed in white adipose tissue (WAT), and its activation has been found to exert anti-lipolytic activity by inhibiting cAMP production and adenylate cyclase action (1). A2BR activation has been demonstrated to inhibit lipogenesis. Notably, A2BR deficiency in mice has been reported to cause WAT inflammation, accompanied by an increase in leptin level (1). Additionally, A2BR expression was observed to be down-regulated in visceral adipose tissue of diet-induced mice (19). Moreover, the A2BR agonist BAY-60–6583 was shown to reduce high-fat diet-induced inflammation in mice by decreasing the level of interleukin (IL)-6 (1). Collectively, these findings provide the evidence for the importance of A1R and A2R in modulating adipogenesis and lipolysis of adipose tissue.

Concerning P2 receptors, lines of evidence have suggested P2X7R, P2Y1R, P2Y2R, P2Y4R, P2Y11R, P2Y13R and P2Y14R are important regulators in various pathogenic abnormality of adipose tissue. P2Y1R and P2Y11R promotes adipogenic differentiation of mesenchymal stem cells (hMSCs) (1). Activation of P2Y13R and P2Y14R inhibits the differentiation of adipocytes of adipose tissue or bone marrow (1). P2Y2R plays a key role in the development of dietary obesity by altering the production of adipokine and lipid (20). Recent studies have shown that P2Y2R activation promotes adipogenesis and expands adipose tissue via regulating leptin level and the extracellular regulated protein kinase (ERK) signalling pathway (1). P2Y1R activation promotes the proliferation and differentiation of adipocytes (1). P2Y2R induces the release of fatty acids and enhances glucose metabolism (21). P2X4R promotes WAT browning by increasing the expression of UCP-1, leading to an increase in energy consumption (1). P2Y11R increases the appetite and food intake (1). P2Y13R promotes the liver uptake of high-density lipoprotein protein and lipid components (22). Adipocyte P2Y14R enhances the activities of key lipolysis enzymes, including adipose triglyceride lipase and hormone-sensitive lipase, thereby exerting lipolytic effects (22). P2X7R activation up-regulates energy expenditure (23) and promotes adipogenesis (1), while P2X7R inhibition causes the abnormal lipid accumulation and increases fat mass (24). Male P2X7R knockout mice exhibits the decreased total cholesterol level and body weight (25).

### 2.2 Purinergic signalling and nerve system in obesity

Central nervous system is critically important in governing energy balance. Activation of agouti-related peptide (AgRP) neurons in the arcuate nucleus of the hypothalamus (ARH) has been reported to promote food intake by modulating appetite (26). The results showed that P2Y6R was expressed in AgRP neurons. A comprehensive study showed that activating P2Y6R through a central application of UDP increased the emission rate of AgRP and feeding in mice (27). Pharmacological inhibition of P2Y6R in AgRP neurons using MRS2578 was found to decrease food intake in mice (27). Moreover, high-fat diet-induced obesity mice had overexpressed A1R in neurons of the hypothalamic paraventricular nucleus (PVN) and overhigh appetite with large body weight (28). In obese mice, a high level of adenosine occurred in cerebrospinal fluid, plasma and hypothalamus (28). Among all adenosine receptors, only A1AR was upregulated in the hypothalamus of obese mice (28), suggesting the primary role of A1AR in obesity. Activation or overexpression of A1AR in the paraventricular nucleus of the hypothalamus has been linked to the increased appetite (28).

### 2.3 Purinergic signalling and inflammatory response in obesity

High free fatty acids (FFAs) have been suggested to be important stimulators for inflammation (29). Interestingly, we have previously showed the important effects of P2X4R on the control of inflammatory responses in RAW264.7 macrophages induced by high FFAs (13). High levels of FFAs were shown to increase the P2X4R expression, which further enhanced the phosphorylation of extracellular ERK and the cytosolic Ca^2+^ concentration, stimulated tumour necrosis factor-α (TNF-α) and iNOS production as well as the release of TNF-α and NO in RAW264.7 macrophages (13). Our findings demonstrated the essential role of P2X4R in the TNF-α and NO release due to elevated FFAs in RAW264.7 cells.

### 2.4 Purinergic signalling and cardiovascular system in obesity

Cardiovascular disease is a main cause of death worldwide (30). Interestingly, our previous study has shown high-fat diet induced P2X7R activation, triggering the nuclear factor kappa-B signalling pathway and cardiomyocyte apoptosis (14). P2X7R activation plays a primary role in the exacerbated myocardial ischemia-reperfusion heart injury in high-fat diet rats via the nuclear factor kappa-B signalling pathway (14). In addition, we found that high FFAs induced the increased expression of P2X7R mRNA and protein in human umbilical vein endothelial cells (HUVECs) (15). The increment of P2X7R expression may be associated with FFA-induced ATP release and reactive oxygen species (ROS) production in HUVECs. Inhibition of P2X7R reversed TNF-α expression caused by high FFAs in HUVECs (15), thus protecting from atherosclerosis.

### 2.5 Purinergic signalling and liver in obesity

NAFLD is a clinical manifestation of metabolic syndrome (31). NAFLD is characterized by the varying degrees of liver damage and inflammation (31). In morbidly obese individuals (BMI > 40 kg/m^2^), the large amount of visceral adipose tissue may contribute to ectopic deposition of lipids in the liver, leading to a high prevalence of NAFLD (31). NAFLD progresses to non-alcoholic steatohepatitis (NASH), a severe form of NAFLD linked to liver damage (32). There are strong associations between NASH and obesity (32, 33). Recent researches have shown the impacts of purinergic signalling on the liver steatosis and inflammation in obesity. In the NASH disease model, the enhanced P2X7R expression was observed in hepatocytes, liver sinusoidal endothelial cells and Kupffer cells, which facilitated the liver injury progression (34). P2X7R activation in Kupffer cells enhanced monocyte chemotactic protein-2 (MCP2) production and TNF-α in carbon tetrachloride-mediated steatohepatitis in obese mice (34). Furthermore, adenosine A2AR was also reported to exert a protective role in NAFLD and inflammation (35, 36). Global and/or myeloid cell-specific A2AR disruption evaluated the severity of high-fat diet-induced hepatic inflammation and steatosis (35). A2AR deficiency dramatically increased the abundance of sterol regulatory element binding protein 1c (SREBP1c) in liver and SREBP1c transcriptional activity in mouse hepatocytes (35), suggesting that A2AR disruption stimulates SREBP1c expression and transcriptional activity and accounts for the increased inflammation and lipogenic events in NAFLD.

### 2.6 Purinergic signalling and renal in obesity

Lipotoxicity caused by a high saturated fat intake induces glomerulosclerosis and tubulo-interstitial damage, which is a vital risk factor for the development of chronic kidney disease (5, 37). It is proposed that the inflammation is a significant mechanism linking lipotoxicity to renal damage (7, 11). ATP and adenosine act as paracrine factors and participate in various pathophysiological functions in the kidney by activating P2 receptor and P1 receptor, respectively (38–40). In the podocytes in the renal cortex, there were increased expressions of P2X7R and NOD-like receptor thermal protein domain associated protein 3 (NLRP3) inflammasome components (41). Activation of P2X7R is necessary for NLRP3 inflammasome activation in a high-fat diet-induced mouse model (41). P2X7R antagonist A438079 or KN-62 significantly attenuated the activation of NLRP3 inflammasome, suggesting that the activated P2X7R/NLRP3 inflammasome may be involved in the podocyte damage of obesity-related glomerulopathy (41, 42). These data support the participation of P2X7R in the process driving “obesity” renal inflammation and injury via P2X7R/NLRP3 inflammasome signalling.

## 3 Purinergic signalling and natural plant extracts

### 3.1 Natural plant extracts ameliorate atherosclerosis by regulating the metabolism of adenine nucleotides

Quercetin is abundant in tea, vegetables, and fruits, such as onions, cucumber, sweet potato, beans, apples and berries (12). Studies have suggested that quercetin prevents high-fat diet-induced obesity via modulating lipogenesis in mice (12). Quercetin inhibited the adipogenesis in 3T3-L1 preadipocytes via stimulating the AMP-activated protein kinase (AMPK) pathway (43). It has been shown that quercetin has effects on regulating ENTPDase 1 and EADA (44). ENTPDase 1, a most widely studied ecto-nucleotidase, which converts ATP and ADP to AMP. EADA converts adenosine into inosine, regulating adenosine receptor engagement (8). Pretreatment with quercetin was indicated to suppress the ENTPDase and EADA activities and reduce the serum ATP and ADP levels, therefore suppressing of inflammatory cell responses and atherosclerosis (45).

### 3.2 Natural plant extracts ameliorate overhigh appetite and atherosclerosis by P1 receptors

Caffeine is an active ingredient in tea, soft drinks and coffee (46). Caffeine is a stimulant and antagonist of adenosine receptors (47). Consumption of caffeine has been associated with the long-term reduction of body weight (48). A1R is widely distributed in the human body and has a particularly high expression in the brain (49). The overexpression of A1R in the neurons of PVN and the increased adenosine level in the cerebrospinal fluid have associations with high body weight in the hyperphagic mice (49). Central or peripheral administration of caffeine suppresses appetite and promotes energy expenditure via inhibiting A1Rs on PVN oxytocin neurons, thus reducing the body weight gain of high FFAs-induced mice (49). Consumption of caffeinated coffee has been demonstrated to diminish weight gain in human (50, 51).

Resveratrol is a natural phytoalexin with anti-atherosclerosis effects. It is extracted from grapes, mulberries, peanuts and rhubarb, which are always applied in dietary supplements (52). Lipotoxicity adds the risk to the endothelial dysfunction and atherosclerosis (3). Pharmacological blocking of A2A receptor by ZM-241385 eliminated the beneficial influences of resveratrol on cholesterol efflux, suggesting that resveratrol may have a key effect on A2A receptor (53). Resveratrol causes an increase in plasma adenosine levels and blood nucleosides (53). Recent study has shown that resveratrol enhances human apolipoprotein A-I activity and high-density lipoprotein-mediated efflux and downregulates oxidized low-density lipoprotein uptake via controlling the major proteins participated in cholesterol transport (53). It has also been demonstrated that resveratrol enhances the activation of A2A receptor and the elimination of cholesterol in cells by modifying the expression of 27-hydroxylase and member 1 of the ATP-binding box subfamily (ABCA1) (53). These researches suggest resveratrol may act as an adenosine agonist, which has the potential to inhibit the abnormal accumulation of lipids, thus alleviating atherosclerosis.

### 3.3 Natural plant extracts ameliorate hepatic steatosis, atherosclerosis and inflammation by P2 receptors

Puerarin, an isoflavone glycoside, is a major active ingredient derived from pueraria lobata. It shows diverse benefits, such as anti-inflammation (16). Maladaptive macrophage activation due to high plasma FFA levels promotes inflammation (13). Under high FFAs condition, we found puerarin could reduce high concentration FFA-elevated P2X4R expression in RAW264.7 macrophages (13). It inhibited high concentration FFA-evoked [Ca^2+^]_i_, ERK phosphorylation, the mRNA expression of TNF-α and iNOS (13). Simultaneously, puerarin inhibited the release of TNF-α and NO induced by high FFA concentration (13). P2X4R selective antagonist 5-BDBD reversed such a high FFA-induced inflammatory activity (13). These findings suggest puerarin may protect against the inflammatory process associated with high plasma FFA levels via P2X4R-mediated inflammation.

Polyphenols, such as quercetin and resveratrol, have been studied for their potential to prevent and alleviate obesity-related metabolic diseases (45). These molecules are available in the form of capsules or pills and can be taken as dietary supplements. P2Y2R deficiency has been reported to alleviate NAFLD by regulating liver lipid metabolism by reducing the levels of aspartate aminotransferase, alanine aminotransferase, and fatty acid synthesis mediators including differentiated CD36 clusters, stearoyl-CoA desaturase 1 and fatty acid synthetase (21). The combination of resveratrol and quercetin reduced P2Y2R expression and ameliorated high-fat-induced hepatic steatosis and NAFLD by enhancing fatty acid oxidation via P2Y2R/AMPK pathway (54). Moreover, our previous study on differential PC12 cells has shown trans-resveratrol could reduce high FFA-induced IL-6 release by inhibiting P2X7R/P38 mitogen-activated protein kinase (MAPK) pathway (17). These data indicate resveratrol and/or quercetin could attenuate lipid metabolism and neuro-inflammation via modulating the expression and the activity of P2Y2R or P2X7R.

Evodiamine is a natural alkaloid mainly extracted from fruits of evodia rutaecarpa (15). Under the high FFA condition, NO formation was obviously reduced, while ROS production and P2X7R expression were enhanced in HUVECs (15). Increased P2X7R expression activated ERK1/2, stimulated the release of inflammatory cytokines such as IL-1β and TNF-α and caused endothelial damage. Evodiamine reversed these harmful changes induced by high FFAs mainly by blocking P2X7R activity (15). Together, evodiamine may exert antioxidative and anti-inflammatory effects, thus protecting from atherosclerosis.

## 4 Conclusion

**Figure 1:**
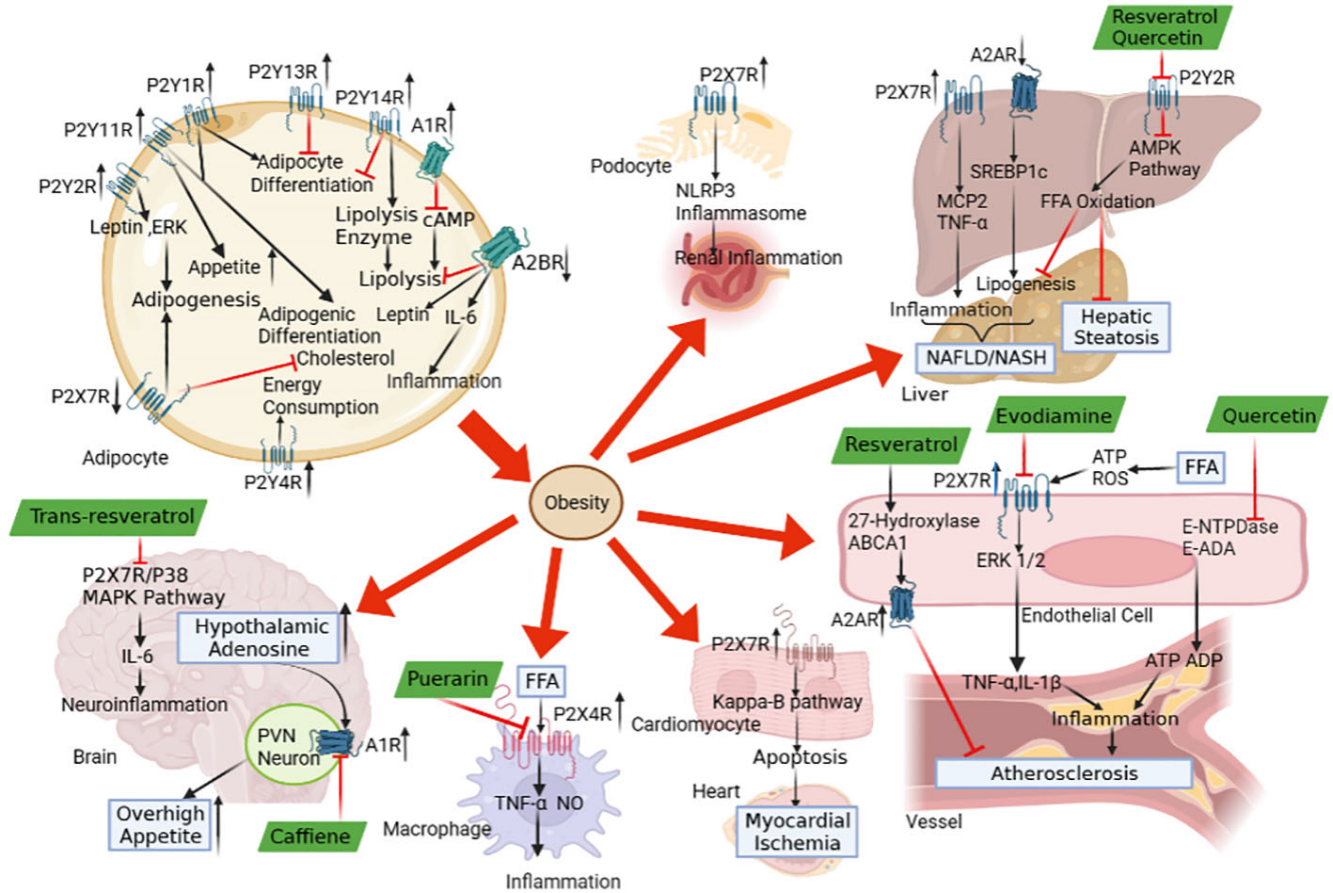
The role of purinergic signalling in obesity-associated end-organ damage and the therapeutic potential of plant natural extracts. The abnormal expressions of P1 receptors and P2 receptors in adipose tissue lead to adipogenesis, adipogenic differentiation and inflammatory response, which contributes to the development of obesity. The purinergic signalling may be regulated by the pathological conditions in obesity, which triggers the end-organ damage, including overhigh appetite, myocardial ischemia, atherosclerosis, inflammation, NAFLD, hepatic steatosis and renal inflammation. The natural plant extracts, including caffeine, puerarin, resveratrol/trans-resveratrol, evodiamine and quercetin, modulate P1 receptors, P2 receptors and ecto-nucleotidase to alleviate obesity-associated end-organ damage via purinergic signalling.

In conclusion, we and others provided much evidence that purinergic signalling affects a wide range of obesity end-organ damage, including overhigh appetite, myocardial ischemia, inflammation, atherosclerosis, NAFLD, hepatic steatosis and renal inflammation (**Figure 1**). Several research papers have denoted plant natural extracts may have therapeutic potential against the atherosclerosis, inflammation, NAFLD and hepatic steatosis via purinergic signalling (**Figure 1**). It should be noted that atherosclerosis, inflammation, NAFLD and hepatic steatosis are just a few of the numerous obesity-related end-organ damage that we and others have demonstrated to be protected by plant natural extracts. With plant natural extracts coming into focus as potential new therapeutic strategies in obesity, more and more obesity-related end-organ damage may be evident to be protected by plant natural extracts via purinergic signalling. Actually, coffee popularity is growing in the official medicine and self-treatment. Consumption of caffeinated coffee has been shown to alleviate obesity in human and animal model. In this respect, coffee may be used as promising candidate for prevention and treatments of obesity. However, there is limit literature available on the therapeutic concentrations of coffee or its bioactive compounds to promote obesity. Thus, more studies are needed to address the safe dose and the limitations of coffee in the prevention and treatment of obesity. As for the other plant natural extracts as drug candidates, they will have to prove benefit in future clinical studies. Further studies on the mechanism and the regulatory efficiency of plant extracts’ role in obesity are prospected to provide new nutritional strategies for the treatment of obesity.

## Author contributions

HX conceived the study. HC, YW, JL, YD, CX, ZT and HY performed a literature search and wrote sections of the manuscript. HX, HC and YH critically revised the manuscript and wrote section of the manuscript. All authors critically reviewed and approved the manuscript.
